# IL-10 Genetic Polymorphisms Were Associated with Valvular Calcification in Han, Uygur and Kazak Populations in Xinjiang, China

**DOI:** 10.1371/journal.pone.0128965

**Published:** 2015-06-03

**Authors:** Yong An, Yong-Tao Wang, Yi-Tong Ma, Muhuyati Wulasihan, Ying Huang, Dilare Adi, Yi-Ning Yang, Xiang Ma, Xiao-Mei Li, Xiang Xie, Ding Huang, Fen Liu, Bang-Dang Chen

**Affiliations:** 1 Department of Cardiology, First Affiliated Hospital of Xinjiang Medical University, Urumqi, 830054 P.R. China; 2 Xinjiang Key Laboratory of Cardiovascular Disease Research, Urumqi, 830054 P.R. China; Cincinnati Children's Medical Center, UNITED STATES

## Abstract

**Objective:**

Valvular calcification occurs via ongoing endothelial injury associated with inflammation. IL-10 is an anti-inflammatory cytokine and 75% of the variation in IL-10 production is genetically determined. However, the relationship between genetic polymorphisms of IL-10 and valvular calcification has not been studied. The objective of this study was to investigate the association between valvular calcification and IL-10 genetic polymorphisms in the Han, Uygur and Kazak populations in China.

**Patients and Methods:**

All of the participants were selected from subjects participating in the Cardiovascular Risk Survey (CRS) study. The single nucleotide polymorphisms (SNPs) rs1800871 and rs1800872 of the IL-10 gene were genotyped using the polymerase chain reaction-restriction fragment length polymorphism (PCR-RFLP) method. Three independent case-control studies involving the Han population, the Uygur population and the Kazak population were used in the analysis.

**Results:**

For the Han and Kazak populations, rs1800871 was found to be associated with valvular calcification in the recessive model, and the difference remained statistically significant following multivariate adjustment (p<0.001, p=0.031, respectively). For the Han, Uygur and Kazak populations, rs1800872 was found to be associated with valvular calcification in the dominant model, and the difference remained statistically significant following multivariate adjustment (p<0.001, p=0.009, and p=0.023,respectively)

**Conclusion:**

Both rs1800871 and rs1800872 of the IL-10 gene are associated with valvular calcification in the Han and Kazak populations in China. Rs1800872 is also associated with valvular calcification in the Uygur population.

## Introduction

Valve calcification, that is aortic valve calcification or mitral annular calcification (MAC), is a common finding in elderly individuals [1]. Although valve calcification was thought to be a passive, degenerative process with generally benign conditions, recent evidence indicates the opposite; instead, it is considered to be actively regulated [2,3]. Otto et al. [4] demonstrated that the presence of aortic valve calcification was associated with an increase of approximately 50 percent for the risk of both death from cardiovascular (CV) causes and a new myocardial infarction. Data from the Framingham Heart Study showed that every 1-mm increase in the MAC was associated with a 10% increased risk of an incident CV disease [5]. Additional studies have shown that aortic valve calcification and MAC are significantly associated with CV morbidity and mortality [6–9].

The etiology and pathogenesis of valvular calcification are likely to comprise a multifactorial disorder, such as endothelial dysfunction, lipid accumulation or inflammatory cell infiltration. The inheritance of several susceptibility genes and multiple environmental determinants are also thought to increase the risk for developing valvular calcification [10–11]. Inflammation is a prominent feature of valve calcification and plays an important role in the development of valvular calcification [12]. Therefore, many genes that are involved in the process of inflammation have been considered to be candidate genes for valvular calcification.

IL-10 is an anti-inflammatory cytokine that is secreted by various cells, and it inhibits TNF-α production by activated macrophages [13]. Moreover, many epidemiological studies have confirmed that TNF-α levels have a strong relationship with valvular calcification [14,15]. In general, TNF-α causes nuclear translocation of the transcription factor nuclear factor-kB (NF-kB) and consequently, accelerates the production of BMP2, which activates alkaline phosphatase through the Runx2/Dlx5 pathway, resulting in valvular calcification [16]. Accordingly, the activity of IL-10 may be associated with the body’s susceptibility to valvular calcification.

The human IL-10 gene is located on chromosome 1 (1q31–1q32) and is composed of 5 exons and 4 introns. Twin studies and family studies have suggested that 75% of the variation in the production of IL-10 is determined by genes [17], and certain polymorphisms may alter the susceptibility to valvular calcification. Moreover, several related studies have demonstrated that polymorphisms in the IL-10 gene have an impact on the patients with severe aortic stenosis undergoing aortic valve replacement surgery in Germans and Canadians [14,18]. However, the relationship between IL-10 gene polymorphisms and valvular calcification remains unclear in the Han, Uygur and Kazak populations of western China.

We conducted this study to explore the relationship between the polymorphisms of the IL-10 gene and valvular calcification in the Han, Uygur and Kazak populations in western China.

## Materials and Methods

### Ethical approval of the study protocol

This study was approved by the Ethics Committee of the First Affiliated Hospital of Xinjiang Medical University (Xinjiang, China). It was conducted according to the standards of the Declaration of Helsinki. All of the patients provided written informed consent and explicitly provided permission for DNA analyses, as well as for the collection of relevant clinical data.

### Subjects

All of the participants were selected from the Cardiovascular Risk Survey (CRS) study; a detailed description of the study population and the methods used was presented previously [19,20]. Briefly, The CRS study is a multi-ethnic, community-based, cross-sectional study designed to investigate the prevalence, incidence and risk factors for cardiovascular diseases and to determine their genetic and environmental contributions to atherosclerosis, coronary artery disease (CAD) and other heart diseases in Chinese Han, Uygur, and Kazakh populations in Xinjiang in West China. The timeframe of the study was from October 2007 to March 2010. The recruited study subjects (aged ≥35 years) were from seven cities in Xinjiang Province: Urumqi, Kelamayi, Hetian, Zhaosu, Fukang, Tulufan, and Fuhai. The staff conducted surveys in each household and administered questionnaires. Information regarding the demographics, socioeconomic status, dietary habits, and the medical history of each participant was collected. Overall, the CRS included 14 618 participants (5757 Hans, 4767 Uygurs, and 4094 Kazakhs).

A total of 1065 participants (605 Hans, 192 Uygurs, and 268 Kazakhs) with complete data were enrolled in the present study. Of the 1065 subjects, 334 participants diagnosed with valvular calcification were screened for the present study as part of the patient population. Simons ACUSON Cypress portable color Doppler ultrasound systems were used for echocardiography and interpreting the images and the patients were categorized on the basis of the presence or absence of an echocardiographically determined aortic valve calcification and/or MAC. The aortic valve calcification was defined as a focal area of echogenicity and thickening of the aortic valve leaflets on the long- or short-axis views with or without stenosis. MAC was defined as an intense echogenicity at the junction of the atrioventricular groove and the mitral valve leaflets in the parasternal and apical views [21–22]. Hypertension was defined as self-reported use of antihypertensive medication within the past 2 weeks or an average systolic blood pressure ≥ 140 mm Hg, an average diastolic blood pressure ≥ 90 mm Hg, or both. Diabetes mellitus was defined according to the World Health Organization (WHO) criteria. Briefly, diabetes was defined as fasting plasma glucose ≥ 7.0 mmol/L, the use of insulin or oral hypoglycemic agents, or a self-reported history of diabetes. Smoking was classified as smokers (including current or ex-smokers) or non-smokers. Patients were excluded if they had congenital heart disease, bicuspid aortic valve, syphilitic heart disease, rheumatic heart disease, chronic renal insufficiency or abnormal calcium and phosphorus metabolism. For each valve calcification patient group, we selected healthy participants matched for ethnicity, sex, and age as the controls. The control subjects were also selected from the CRS. These individuals did not have any relevant valvular abnormalities in their echocardiograms or a history of valvular heart disease [19,20].

### Biochemical analyses

The blood samples were collected in vacutainer tubes containing EDTA from an antecubital vein in the morning after an overnight fasting period. All of the collected samples were transported on dry ice at prearranged intervals to the Xinjiang coronary artery disease VIP laboratory. The serum total cholesterol (TC), triglyceride (TG), fasting glucose, low density lipoprotein (LDL), high density lipoprotein (HDL), blood urea nitrogen (BUN), creatinine (Cr) and uric acid concentrations were measured by the Clinical Laboratory Department of the First Affiliated Hospital of Xinjiang Medical University using a biochemical analyzer (Dimension AR/AVL Clinical Chemistry System, Newark, NJ, USA) [19–20].

### IL-10 genotyping

**Fig 1:**
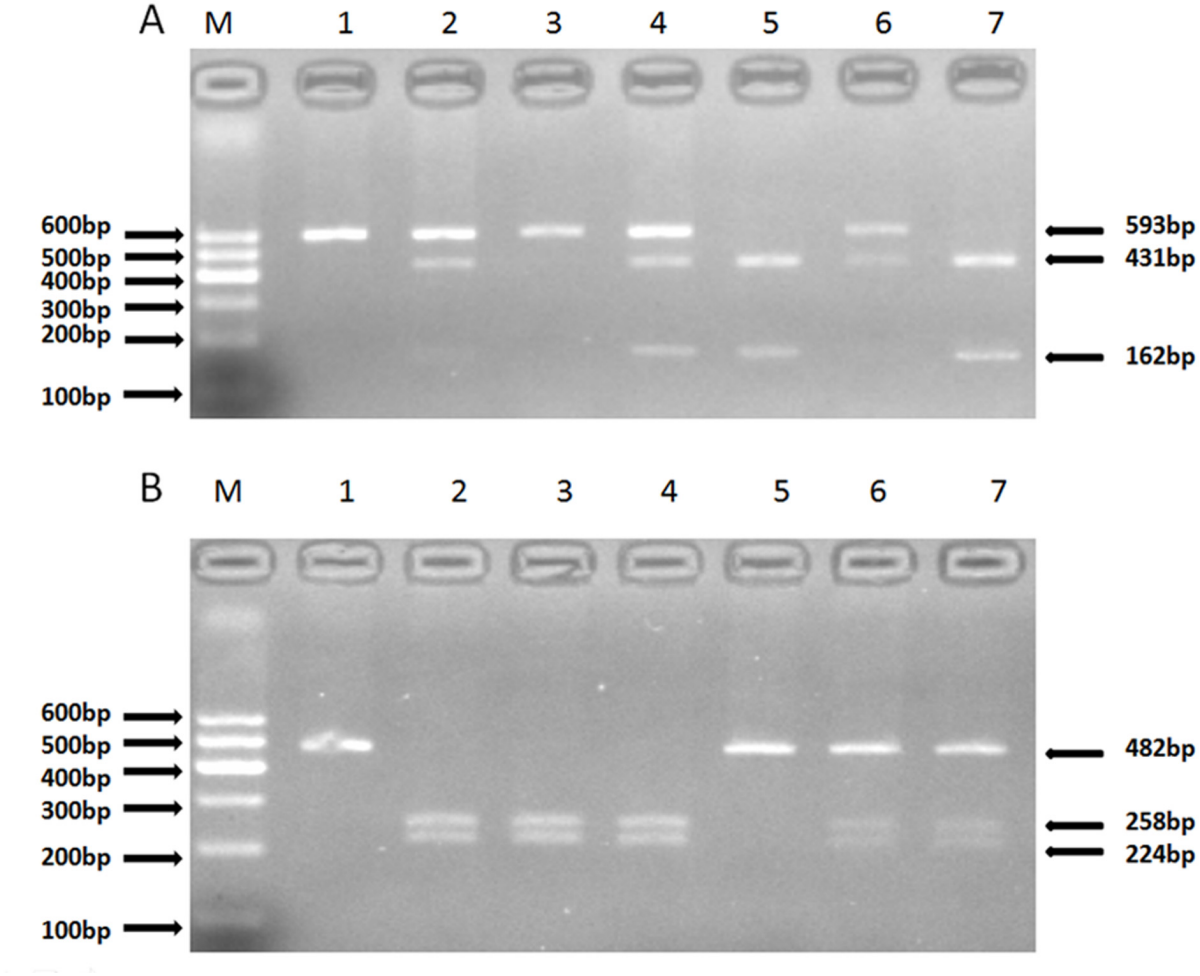
The restriction fragment length polymorphism analysis to determine the genotype. A. For rs1800871, the TT genotype shows one 593-bp band (1 and 3); the CC genotype shows two bands at 431 bp and 162 bp (5 and 7); and the CT genotype shows three bands at 593 bp, 431 bp and 162 bp (2, 4 and 6). B. For rs1800872, the CC genotype shows one 482-bp band (1 and 5); the AA genotype shows two bands at 258 bp and 224 bp (2, 3 and 4); and the AC genotype shows three bands at 482 bp, 258 bp and 224 bp (6 and 7).

There are 189 SNPs for the human IL-10 gene listed in the National Center for Biotechnology Information SNP database (http://www.ncbi.nlm.nih.gov/SNP). Using Haploview 4.2 and the International HapMap Project website phase I & II database (http://www.hapmap.org), we obtained two tagging SNPs (rs1800871, rs1800872) using a minor allele frequency (MAF) ≥0.05 and linkage disequilibrium patterns with an r2≥0.8 as a cutoff. Genomic DNA was extracted from the peripheral blood leukocytes using a DNA extraction Kit (Beijing Biotech Company Limited, Beijing, China). The genotyping in the present study was confirmed via polymerase chain reaction (PCR)-restriction fragment length polymorphism (RFLP) analysis. The sequencing primers were designed using Primer Premier 5.0. Synthesis of the primers was undertaken by Shanghai Genery Biological Technology Company Limited (Shanghai, China). The PCR amplification was performed using 25 μL of 2*powder Taq PCR master mix (Beijing Biotech, Beijing, China), 50 ng of genomic DNA, 21 μL of distilled water, and 1 μL of each forward and reverse primer in a 50-μL final reaction volume. The thermal cycling conditions were as follows: an initial denaturation step at 95°C for 5 min; 35 cycles of 95°C for 30 s, 51.3°C for 30 s and 72°C for 45 s, which was followed by a final extension step of 72°C for 10 min. The thermal cycling was performed using the GeneAmp 9700 system (Applied Biosystems). The digestion of the PCR products by restriction enzymes was performed according to the manufacturer’s instructions. The primer pair sequences, annealing temperatures, resulting fragments and restriction enzymes for the two SNPs are detailed in Table 1. The digested products were analyzed on 3% agarose gels and stained with ethidium bromide (Fig 1). A total of 10% of the genotyped samples were duplicated, and there was at least one positive and one negative control per 96-well DNA plate in our test. We obtained 100% concordance between the genotyped duplicate samples for each of the SNPs.

**Table 1:** The primer sequences for each SNP.

SNPs	Polymerase chain reaction primers	Denaturation temperature	Products length	Restriction enzyme
rs1800871	Sense5′AAACTTTAGACTCCAGCCACA3′	51.3°C	593	Msl I
	Antisense5′TGCCCATTCCAGAATACAA3′			
rs1800872	Sense5′TCTGTGCCTCAGTTTGC3′	51.3°C	482	Afa I
	Antisense5′CTGTGGGTTCTCATTCG3′			

## Statistical Analysis

The data analysis was performed using SPSS version 17.0 for Windows (SPSS Inc., Chicago, IL, USA). The Hardy-Weinberg equilibrium was assessed via X2 analysis. The measurement data are shown as the means±SD, and the differences between the valvular calcification subjects and the control subjects were assessed using an independent-sample t-test. Differences in the enumeration data, such as the frequencies of smoking, drinking, hypertension and IL-10 genotypes between the valvular calcification patients and the control subjects were analyzed using the chi-square test. Additionally, logistic regression analyses with effect ratios (odds ratio [OR] and 95% CI) were used to assess the contribution of the major risk factors. A P value < 0.05 was considered to be statistically significant.

## Results

In total, 334 valvular calcification patients (cases) and 731 controls were enrolled in this study. Table 2 shows their demographic and clinical characteristics. Among the Han subjects, the cases and controls differed significantly with regards to hypertension; diabetes mellitus (DM); systolic blood pressure (SBP) and the plasma total cholesterol (TC) and low density lipoprotein (LDL) (all P<0.05) concentrations.. Among the Uygur subjects, the following variables were significantly different between the two groups: smoking status; drinking status; SBP; DBP; and the serum TC and LDL concentrations (all P<0.05). Among the Kazak subjects, hypertension; BMI; SBP; DBP; and the serum TC, LDL and Cr concentrations were significantly higher in the subjects with valvular calcification than in the controls (all P<0.05).

**Table 2:** Demographic and clinical characteristics of study participants.

	Han			Uygur			Kazak		
	Valvular calcification	Control	P value	Valvular calcification	Control	P value	Valvular calcification	Control	P value
Number(n)	194	411		59	133		81	187	
Age,years	67.58(8.21	67.22(8.93	0.629	67.30(10.8	67.05(10.24	0.873	67.32(7.74)	66.47(8.93)	0.454
Sex,female,	83(42.8)	177(43.1)	0.948	37(62.7)	63(47.4)	0.06	44(54.3)	93(49.7)	0.49
Hypertensio	119(61.3)	201(48.9)	0.004	36(61)	69(51.9)	0.273	69(85.2)	104(55.6)	<0.001
Diabetes, n	34(17.5)	46(11.2)	0.032	4(6.8)	6(4.5)	0.5	4(4.9)	6(3.2)	0.493
Smoking, n	55(28.4)	101(24.6)	0.322	10(16.9)	4(3)	<0.001	27(33.3)	47(25.1)	0.168
Drinking, n	20(10.3)	47(11.4)	0.68	24(40.7)	13(9.8)	<0.001	5(6.2)	9(4.8)	0.646
BMI,kg/m^2^	25.84(3.71	25.85(3.59	0.973	24.87(4.12	25.27(4.52	0.487	28.02(4.79)	26.44(4.18)	0.007
SBP,mm H	144.35(19.1	139.81(18.5	0.006	146.24(30.3	135.89(19.5	0.005	164.83(29.61)	148.48(25.87)	<0.001
DBP,mm H	86.36(14.87	86.64(16.18	0.839	88.19(19.84	81.89(13.28	0.011	110.64(22.75)	90.41(19.68)	<0.001
Glucose,m	5.78(2.04)	5.48(1.81)	0.073	4.69(1.35)	4.64(1.15)	0.799	5.41(2.03)	5.29(2.01)	0.637
TG,mmol/L	1.61(1.19)	1.65(1.36)	0.746	1.58(0.93)	1.43(0.85)	0.282	1.17(0.57)	1.30(1.05)	0.327
TC,mmol/L	4.67(1.11)	4.42(1.22)	0.019	4.89(1.16)	4.38(1.07)	0.003	5.21(1.07)	4.79(1.07)	0.004
HDL,mmol/	1.24(0.46)	1.25(0.52)	0.901	1.27(0.40)	1.26(0.42)	0.877	1.30(0.43)	1.28(0.42)	0.684
LDL,mmol/	2.94(1.00)	2.69(0.84)	0.001	3.08(1.01)	2.71(0.81)	0.007	3.08(0.95)	2.81(1.00)	0.037
UA,umol/L	314.62(93.6	300.67(81.0	0.061	260(68.08)	252.68(71.9	0.51	268.28(86.43)	272.02(74.99)	0.721
SCr,umol/L	76.46(23.92	76.56(40.75	0.975	80.06(30.24	72.73(26.36	0.091	76.65(17.97)	68.94(19.96)	0.003
BUN,mmol/	5.36(1.66)	5.25(1.59)	0.447	5.78(1.63)	6.18(3.19)	0.362	5.18(1.77)	4.93(1.56)	0.257

Note: 1 mm Hg = 0.133 KPa; Glucose: 1mmol/L = 18 mg/dl; TG: 1mmol/L = 88.6 mg/dl; TC: 1mmol/L = 38.7 mg/dl; HDL: 1mmol/L = 38.7 mg/dl; LDL: 38.7 mg/dl; UA: 1umol/L = 0.0168 mg/dl; SCr: 1umol/L = 0.0113 mg/dl; BUN: 1mmol/L = 2.8 mg/dl;Normal ranges for laboratory values: SBP: 90–139 mm Hg; DBP: 60–89 mm Hg; Glucose: 3.9–6.1 mmol/L; TG: 0.56–1.7 mmol/L; TC: 2.8–5.72 mmol/L; HDL: 1.16–1.55 mmol/L; LDL: 2.7–3.1 mmol/L; UA: 208–428 umol/L; SCr: 53–115 umol/L; BUN: 2.9–8.2 mmol/L.

Table 3 shows the distribution of the genotypes and alleles for the two SNPs of the IL-10 gene in the Han, Uygur and Kazak populations. The genotype distributions for each of the SNPs were in good agreement with the predicted Hardy-Weinberg equilibrium values in the three ethnicities (P>0.05). For the Han population, the distribution of the rs1800871 genotypes, the recessive model (TT vs CC + CT), the additive model (CT vs CC+TT) and the allele frequency showed significant differences between the cases with valvular calcification and the control subjects (p = 0.004, p = 0.001, p = 0.015 and p = 0.001, respectively). For the Uygur population, there was no significant difference between the cases with valvular calcification and the control subjects in regards to the distribution of the rs1800871 genotypes, the dominant model (CC vs TT +CT), the recessive model (TT vs CC+ CT), the additive model (CT vs CC+TT) or the allelic distribution (p = 0.714, p = 0.526, p = 0.785, p = 0.42 and p = 0.796, respectively). For the Kazak population, the recessive model (TT vs CC + CT) of rs1800871 showed a significant difference between the cases with valvular calcification and the control subjects (p = 0.038). For the Han population, the C allele of rs1800871 was significantly higher in the controls than in the cases with valvular calcification (36.9% vs 27.6%). For the Han and Kazak populations, the recessive model (TT vs. CC + CT) of rs1800871 was significantly lower in the controls than in the cases with valvular calcification (Han: 40.6% vs 55.2%; Kazak: 25.7% vs. 38.3%).

**Table 3:** Genotype and Allele distributions in patients with valvular calcification and control participants.

	Han			Uygur			Kazak		
Variant	Valvular calcification(%)	control(%)	P value	Valvular calcification(%)	control(%)	P value	Valvular calcification(%)	control(%)	P value
rs1800871									
Genotype									
TT	107(55.2)	167(40.6)		13(22)	27(20.3)		31(38.3)	48(25.7)	
CC	20(10.3)	59(14.4)		20(33.9)	39(29.3)		13(16)	40(21.4)	
CT	67(34.5)	185(45)	0.004	26(44.1)	67(50.4)	0.714	37(45.7)	99(52.9)	0.108
Dominant model									
CC	20(10.3)	59(14.4)		20(33.9)	39(29.3)		13(16)	40(21.4)	
CT+TT	174(89.7)	352(85.6)	0.168	39(66.1)	94(70.7)	0.526	68(84)	147(78.6)	0.313
Recessive model									
TT	107(55.2)	167(40.6)		13(22)	27(20.3)		31(38.3)	48(25.7)	
CT+CC	87(44.8)	244(59.4)	0.001	46(78)	106(79.7)	0.785	50(61.7)	139(74.3)	0.038
Additive model									
CT	67(34.5)	185(45)		26(44.1)	67(50.4)		37(45.7)	99(52.9)	
CC+TT	127(65.5)	226(55)	0.015	33(55.9)	66(49.6)	0.42	44(54.3)	88(47.1)	0.275
Allele									
C	107(27.6)	303(36.9)		66(55.9)	145(54.5)		63(38.9)	179(47.86)	
T	281(72.4)	519(63.1)	0.001	52(44.1)	121(45.5)	0.796	99(61.1)	195(52.14)	0.055
rs1800872									
genotype									
CC	64(33)	63(15.3)		31(52.2)	46(34.6)		36(44.4)	55(29.4)	
AA	77(39.7)	163(39.7)		10(16.9)	26(19.5)		18(22.2)	50(26.7)	
AC	53(27.3)	185(45)	0	18(30.5)	61(45.9)	0.056	27(33.3)	82(43.9)	0.056
Dominant model									
CC	64(33)	63(15.3)		31(52.2)	46(34.6)		36(44.4)	55(29.4)	
AC+AA	130(67)	348(84.7)	0	28(47.5)	87(65.4)	0.019	45(55.6)	132(70.6)	0.017
Recessive model									
AA	77(39.7)	163(39.7)		10(16.9)	26(19.5)		18(22.2)	50(26.7)	
AC+CC	117(60.3)	248(60.3)	0.994	49(83.)	107(80.5)	0.67	63(77.8)	137(73.3)	0.435
Additive model									
AC	53(27.3)	185(45)		18((30.5)	61(45.9)		27(33.3)	82(43.9)	
AA+CC	141(72.7)	226(55)	0	41(69.5)	72(54.1)	0.046	54(66.7)	105(56.1)	0.107
Allele									
C	181(46.6)	311(37.8)		80(67.8)	153(57.5)		99(61.1)	192(51.3)	
A	207(53.4)	511(62.2)	0.004	38(32.2)	113(42.5)	0.057	63(38.9)	182(48.7)	0.037

For the Han population, the distribution of the rs1800872 genotypes, the dominant model (CC vs AA + AC), the additive model (AC vs CC+AA) and the allele frequency showed significant differences between the cases with valvular calcification and the control subjects (p<0.001, p<0.001, p<0.001 and p = 0.004, respectively). For the Uygur population, the dominant model (CC vs AA + AC) and the additive model (AC vs CC+AA) for rs1800872 were significantly different between the cases with valvular calcification and the control subjects (p = 0.019, p = 0.046, respectively). For the Kazakh population, the dominant model (CC vs AA + AC) for rs1800872 showed a significant difference between the cases with valvular calcification and the control subjects (p = 0.017). For the Han and Kazakh populations, the A allele of rs1800872 was significantly higher in the control subjects than in the cases with valvular calcification (Han: 62.2% vs 53.4%; Kazakh: 48.7% vs 38.9%). For the Han, Uygur and Kazakh populations, the dominant model (CC vs AA + AC) was significantly higher in the cases with valvular calcification than in the control subjects (Han: 33% vs 15.3%; Uygur: 52.2% vs 34.6%; Kazakh: 44.4% vs 29.4%).

Tables 4 and 5 show the multivariable logistic regression analyses of the major confounding factors for valvular calcification. Table 4: For rs1800871, The odds ratio (OR) for carriers of TT genotype for valvular calcification was 1.797 [95% confidence interval (CI): 1.797–2.536] in Han subjects and 1.795 [95% CI: 1.030–3.129] in Kazak subjects. Following the multivariate adjustments for the confounders, such as the plasma TC, LDL concentrations, Hypertension and DM, the difference remained significant in Han subjects (P<0.001, OR = 1.894, 95% CI: 1.324–2.709); there also exists significance in Kazak subjects (P = 0.031, OR = 1.952, 95% CI: 1.062–3.586) after adjustment of confounders such as the plasma TC, LDL concentrations, Hypertension and BMI. Table 5: For rs1800872, The odds ratio (OR) for carriers of CC genotype for valvular calcification was 1.795 (CI: 1.030–3.129) in Han subjects, 2.094 (CI: 1.122–3.906) in Uygur subjects and 1.920 (CI: 1.119–3.293) in Kazak subjects. Following the multivariate adjustments for the confounders, such as the plasma TC, LDL concentrations, Hypertension and DM, the difference remained significant in Han subjects (P<0.001, OR = 2.565, 95% CI: 1.699–3.874); following adjustments for the plasma TC, LDL concentrations, Hypertension and smoker, the difference remained significant in Uygur subjects (P = 0.009, OR = 2.621, 95% CI: 1.275–5.387); after adjustment of confounders such as the plasma TC, LDL concentrations, Hypertension and BMI, there still exists significance in the Kazak subjects (P = 0.023, OR = 1.981, 95% CI: 1.100–3.570).

**Table 4:** Results of Logistic analysis (rs1800871).

Han				Kazak			
	OR	95% C.I.	P		OR	95% C.I.	P
Recessive model 1	1.797	1.273–2.536	0.001	Recessive model 1	1.795	1.030–3.129	0.039
Recessive model	1.894	1.324–2.709	<0.001	Recessive model	1.952	1.062–3.586	0.031
TC	1.178	1.010–1.373	0.037	TC	1.413	1.081–1.846	0.011
LDL	1.424	1.170–1.732	<0.001	LDL	1.391	1.051–1.841	0.021
Hypertention	1.612	1.119–2.322	0.01	Hypertention	4.091	2.017–8.298	<0.001
DM	1.784	1.077–2.957	0.025	BMI	1.057	0.991–1.127	0.093

Recessive model 1: Unadjusted model; Recessive model: adjusted model.

**Table 5:** Results of Logistic analysis (rs1800872).

Han				Uygur				Kazak			
	OR	95% C.I.	P		OR	95% C.I.	P		OR	95% C.I.	P
Dominant model 1	1.795	1.03–3.129	0.039	Dominant model 1	2.094	1.122–3.906	0.02	Dominant model 1	1.92	1.119–3.293	0.018
Dominant model	2.565	1.699–3.874	<0.001	Dominant model	2.621	1.275–5.387	0.009	Dominant model	1.981	1.100–3.570	0.023
TC	1.199	1.027–1.399	0.021	TC	1.613	1.159–2.244	0.005	TC	1.4	1.066–1.839	0.016
LDL	1.398	1.147–1.704	0.001	LDL	1.312	0.881–1.953	0.181	LDL	1.419	1.072–1.880	0.014
Hypertension	1.495	1.035–2.158	0.032	Hypertension	1.73	0.840–3.561	0.137	Hypertension	4.233	2.081–8.608	<0.001
DM	1.593	0.961–2.640	0.071	Smoking	3.693	2.030–6.721	<0.001	BMI	1.041	0.976–1.110	0.22

Dominant model 1: Unadjusted model; Dominant model: adjusted model.

## Discussion

In the present study, we found that variation in the IL-10 gene is associated with valvular calcification in the Han, Uygur and Kazak populations of Xinjiang (West China). After a multivariate adjustment, there was still a significant difference between the IL-10 gene polymorphisms and valvular calcification. Our study is the first case-control study to investigate the association between the human IL-10 gene and valvular calcification in the Han, Uygur and Kazak populations in Xinjiang, China.

Xinjiang is a multi-ethnic co-populated area. The sixth national census showed that there are 47 ethnicities living in Xinjiang, and 13 of them are confirmed to be native ethnicities, such as the Uygur, Han, Kazak, Hui, Kirgiz, Mongolian, Tajik, Xibe, Manchu, Uzbek, Russian, Daur and Tatar people. Among them, the Uygur people account for 46%, the Han account for 40% and the Kazak account for 7%. We noted that the different ethnicities in Xinjiang may be a confounding factor of the present study and the genetic backgrounds of these different ethnicities may be helpful for explaining the mechanism behind valvular calcification. However, until now, there has been no data regarding the genetic backgrounds of these 13 ethnicities. Miscegenation is very rare in this area and we also excluded those who had a history of miscegenation when selecting the participants for the present study.

The high prevalence of valvular heart diseases, together with its increase with ageing, indicate it a high and increasing burden. The prevalence of valvular calcification were reported between 28% and 46.1% in China [23]. Although there are evidences showed that valvular calcification is likely to occur among members of a family, the genetic factors are still elusive to explain this heritability. Prior genomewide association study conducted by Thanassoulis et al.[24] have found that the SNP rs10455872 in the gene LPA was strongly associated with the presence of aortic valve calcification (OR per minor G allele: 2.05, 95% CI: 1.63–2.57, P = 9.0 ×10^–10^). Importantly, their finding was further replicated in additional white European, African-American, and Hispanic-American cohorts (P < 0.05 for all comparisons) and demonstrated that genetic variation at the LPA locus play an important role in the development of aortic valve calcification by regulating plasma Lp(a) levels. Polymorphisms of other candidate genes have also been demonstrated to be associated with valvular calcification in several research [11]. All this indicated that genetic predisposition are required for the genesis of valvular calcification.

It is well known that IL-10 is an important pleotropic cytokine with anti-inflammation functions, and it is produced by the monocytes and lymphocytes [25–27]. It exerts its anti-inflammatory effect by inhibiting Th1-type responses and monocyte functions via the inhibition of the production of multiple proinflammatory cytokines, such as TNF-α, IL-1α and IL-8 [28–29]. Previous studies have demonstrated that valvular calcification occurs via ongoing endothelial injuries associated with inflammatory cell infiltration [30], and large amounts of TNF-α exist in the extracellular matrix of calcified valve tissue [15,31,32]. Therefore, TNF-α may contribute to the calcification process of the valve. Further research showed that TNF-a promotes monocyte adhesion and foam cell formation by binding to TNFRSF1A, which is expressed in human aortic valve interstitial cells (HAVICs), and may regulate the activation of NF-κB [33]. More studies have proven that TNF–a can promote the expression of BMP2 via the NF-κB pathway [16]. BMP2 is expressed by myofibroblasts and preosteoblasts in the calcific valves, and it can activate alkaline phosphatase (ALP) via the Runx2/Dlx5 pathway, ultimately resulting in calcification [15]. Therefore, the activation of IL-10 may be associated with valvular calcification.

IL-10 production is largely genetically controlled at a transcriptional level [34]. The IL-10 5' flanking region, which controls transcription, is polymorphic, and contains not only two microsatellites between -4000 and -1200 but also three single base-pair substitutions: rs1800871, rs1800872 and rs1800896. There is linkage disequilibrium between the alleles and only three different haplotypes were found in previous studies: GCC, ACC, and ATA. Researches have demonstrated that the GCC/GCC genotype is associated with higher and the ATA haplotype with lower production of IL10 in peripheral blood mononuclear cells than other genotypes as polymorphisms of the haplotype are in close proximity to several transcription factors that may interfere with gene transcription [35].

Research regarding the relationship between the polymorphisms of the IL-10 gene and valvular calcification was first reported by Ortlepp et al [14]. 187 patients with calcific aortic stenosis in different degrees were genotyped in their study (rs1800871: CC: 106, TT: 7, CT: 74; rs1800872: CC: 105, AA: 7, AC: 75; rs1800896: GG: 41, AA: 54, AG: 92) and the G allele of rs1800896, the C allele of rs1800871, and the C allele of rs1800872 were significantly associated with the degree of calcification (P = 0.026, 0.027, and 0.028, respectively). Further more, they demonstrated that the high IL10-producing haplotype (GCC/GCC) was associated with a higher degree of calcification in stenotic aortic valves A replication of the genetic association study by Nathalie Gaudreault et al. [18] found there exist significant difference between the cases with valvular calcification and controls (1800872: A: 29.8% vs 20.2%, C: 70.2 vs 79.8%; 1800896: G: 42% vs 48.4%, A: 58% vs 51.6%). They demonstrated that SNPs (the A allele of rs1800896 and the A allele of rs1800872) tagging the low-producing IL10 haplotype (ATA) was associated with a higher degree of calcification in AS (P = 0.000297 and 6.21×10^–11^). But it did not demonstrate any correlation between the rs1800871 polymorphism and valvular calcification.

In the present study, we genotyped the rs1800871 and rs1800872 polymorphisms in the IL-10 gene and found that they were associated with valvular calcification. For rs1800871, the frequency of the T allele was significantly higher in the cases than in the controls in the Han population. This indicates that the risk of valvular calcification may increase with the presence of the T allele in the Han population. Similarly, the recessive model (TT vs CT+ CC) was significantly higher in the cases than in the controls; even after adjustment for other risk factors, the significant difference still remained. This indicates that the C allele may be protective against valvular calcification. For rs1800872, the frequency of the C allele was higher in the cases than in the controls in the Han and Kazak populations. This indicates that the risk for valvular calcification increases in the presence of the C allele in the Han and Kazak populations. Similarly, the dominant (CC vs AC+AA) model showed a significant difference between the cases and the controls. The significant difference in the dominant model between the two groups still existed after the multivariate adjustment for confounding factors. This indicates that the A allele may be protective against valvular calcification.

Based on the above studies, we believe the association between the polymorphisms of the IL-10 gene and valvular calcification vary as the orientation of the risk alleles was reversed. The reason for this discrepancy is unclear but may be a result of diet, ethnic differences, environmental factors and our relatively small-scale study. Therefore, further large-scale investigations should be carried out on different ethnic populations.

In conclusion, polymorphisms of the IL-10 gene were associated with valvular calcification in the Kazak, Han and Uygur populations of western China. This result may broaden the knowledge of genetic variants and disease-association studies. Additional studies need to be undertaken to clarify the underlying molecular mechanism that associates the IL-10 polymorphisms with valvular calcification.
